# A Cross-Sectional Study of CD163 Expression and CD8-Positive Tumor-Infiltrating Lymphocytes in Glial Tumors at a Tertiary Care Center

**DOI:** 10.7759/cureus.105981

**Published:** 2026-03-27

**Authors:** Aditya Nair, Naval Kishore

**Affiliations:** 1 Department of Pathology, Osmania Medical College, Hyderabad, IND

**Keywords:** cd163, cd8, glial tumors, glioma, immunohistochemistry, tumor-associated macrophages, tumor-infiltrating lymphocytes, tumor microenvironment, who grade

## Abstract

Background and objective

Glial tumors are increasingly recognized as immunologically active neoplasms in which macrophage-rich and lymphocyte-poor microenvironments may influence aggressiveness. Cluster of differentiation 163 (CD163) highlights tumor-associated macrophages, whereas CD8 marks cytotoxic tumor-infiltrating lymphocytes (TILs). This study aimed to evaluate CD163 expression and CD8-positive TILs in glial tumors and examine their association with tumor grade.

Methods

This cross-sectional study included 50 histopathologically confirmed glial tumors. Immunohistochemistry for CD163 and CD8 was performed on formalin-fixed paraffin-embedded tissue sections. CD163 was scored as absent, weak, moderate, or strong, and CD8 infiltration was categorized as low or high. Associations with World Health Organization (WHO) tumor grade and histologic aggressiveness were analyzed using the chi-square test, Fisher's exact test, and Spearman's correlation.

Results

The mean age of the cohort was 42.7 ± 16.1 years, with males accounting for 29/50 (58.0%) and females for 21/50 (42.0%). High-grade gliomas comprised 32/50 (64.0%) cases, while low-grade tumors accounted for 18/50 (36.0%); grade IV tumors were the largest subgroup (20/50, 40.0%). CD163 positivity was observed in 40/50 (80.0%) tumors and increased significantly with tumor grade (rho = +0.59, p < 0.001). CD163 positivity was more frequent in high-grade than in low-grade tumors (30/32 (93.8%) vs. 10/18 (55.6%), p = 0.004). High CD8 infiltration was seen in 15/50 (30.0%) tumors and low CD8 infiltration in 35/50 (70.0%), with CD8 levels declining significantly with increasing grade (rho = -0.33, p = 0.021). Strong CD163 expression with low CD8 infiltration was present in 17/50 (34.0%) tumors and was significantly associated with high-grade lesions (16/32 (50.0%) vs. 1/18 (5.6%), p = 0.004). Moderate-to-strong CD163 expression was associated with necrosis (18/20 (90.0%), p = 0.009) and microvascular proliferation (16/18 (88.9%), p = 0.024).

Conclusions

Higher-grade glial tumors demonstrate a macrophage-dominant and CD8-restricted immune microenvironment. Combined assessment of CD163 and CD8 may serve as a practical indicator of aggressive tumor biology in routine diagnostic practice.

## Introduction

Glial tumors are increasingly understood not merely as neoplasms of disordered neuroepithelial growth but as biologically active ecosystems in which tumor cells and immune elements interact and co-evolve [1,2]. Among these immune components, macrophage-rich infiltrates and cytotoxic T lymphocytes appear especially relevant to tumor behavior, progression, and therapeutic resistance. Accumulating evidence shows that cluster of differentiation 163 (CD163), a marker closely linked to alternatively polarized immunosuppressive macrophages, increases with glioma aggressiveness, is enriched in isocitrate dehydrogenase (IDH)-wild type and mesenchymal tumors, and correlates with adverse survival, stromal activation, angiogenesis, and checkpoint signaling [3,4].

In glioblastoma, the microenvironment is often macrophage-dominant rather than lymphocyte-rich, with CD163-positive cells frequently outnumbering CD8 infiltrates, suggesting that immune suppression may be structurally embedded within high-grade disease [4,5]. Mechanistic studies further indicate that CD163-positive myeloid populations are not passive bystanders; they actively promote T-cell dysfunction through interleukin-10 (IL-10)-driven exhaustion programs and reinforce mesenchymal, wound-healing, and angiogenic phenotypes within the tumor microenvironment [6]. In contrast, CD8-positive tumor-infiltrating lymphocytes (TILs) represent the principal effector arm of anti-tumor immunity, yet their role in gliomas is complex. Some studies suggest that higher CD8 infiltration or enrichment of tissue-resident memory CD8 subsets reflects a more favorable immune contexture, whereas others show that prognosis depends less on absolute CD8 count and more on the relative balance between effector and suppressive immune compartments [7].

Reviews of glioblastoma immunobiology consistently emphasize that macrophage-mediated suppression blunts CD8 function and limits meaningful anti-tumor immunity [8,9]. Despite these advances, most data have been derived from glioblastoma alone, whereas comparative information across the broader spectrum of glial tumors remains limited. Against this background, this study was designed to evaluate CD163 expression and CD8-positive TILs in glial tumors and to examine their relationship with tumor grade.

## Materials and methods

Study design and setting

This was a cross-sectional observational study conducted in the Department of Pathology, Osmania Medical College, Koti, Hyderabad, from January 28, 2024, to July 28, 2025. Ethical approval was obtained from the Institutional Ethics Committee, Osmania Medical College (Ref: IEC/OMC/23118001001D). The study involved both archived and prospectively collected tissue specimens from histopathologically confirmed glial tumors. A cross-sectional tissue-based design was considered appropriate because the objective was to assess the immunohistochemical expression of CD163 and CD8 in tumor samples at a single point in pathological evaluation and to examine their association with tumor grade.

Study population and sampling

A total of 50 cases of glial tumors were included in the study using convenience sampling. All eligible cases received during the study period that met the inclusion criteria were considered for analysis.

Eligibility criteria

Inclusion Criteria

Cases were included in the study if they fulfilled all of the following conditions: histopathologically confirmed diagnosis of a glial tumor on routine microscopic examination; availability of adequate formalin-fixed, paraffin-embedded tissue for immunohistochemical analysis; and presence of sufficient viable and representative tumor tissue to permit reliable evaluation of both CD163 expression and CD8-positive TILs. Cases received during the defined study period and accompanied by the essential clinicopathological details necessary for classification and grading were considered eligible for inclusion.

Exclusion Criteria

Cases were excluded if the tissue sample was scant, inadequate, or poorly preserved for immunohistochemical assessment. Specimens showing extensive necrosis, hemorrhage, crush artifact, or marked autolysis that could compromise interpretation were also excluded. Tumors from patients who had received prior radiotherapy or chemotherapy were excluded to avoid treatment-related alterations in immune-cell infiltration and antigen expression. Non-glial neoplasms, inflammatory lesions, reactive conditions, and other non-neoplastic central nervous system (CNS) lesions were similarly excluded. Cases in which definitive histopathological classification could not be established or in which the tissue was not representative of the tumor were omitted from the analysis.

Histopathological review and tumor grading

All hematoxylin and eosin-stained slides were reviewed by an experienced pathologist. Tumors were classified as glial neoplasms and graded according to accepted World Health Organization (WHO) diagnostic criteria based on histomorphology and available clinical records. For the purpose of this study, the principal clinicopathological variable used for correlation was tumor grade. Histological features, including cellularity, nuclear atypia, mitotic activity, microvascular proliferation, and necrosis, were also recorded when applicable.

Tissue processing and section preparation

Representative tumor blocks were selected from formalin-fixed, paraffin-embedded tissue. Sections were cut at an appropriate thickness and mounted on coated glass slides for immunohistochemistry. The sections were deparaffinized in xylene and rehydrated through graded alcohols. Antigen retrieval was performed using 10 mM sodium citrate buffer at pH 6.0 with heat-induced epitope retrieval at 95 to 100 °C for 20 minutes, followed by cooling at room temperature for 20 minutes and washing with TBST buffer. Standard immunohistochemical staining procedures were then carried out.

Immunohistochemistry

Immunohistochemical evaluation was performed using antibodies against CD163 and CD8. CD163 was used as a marker of tumor-associated macrophages with immunosuppressive phenotypes, whereas CD8 was used as a marker of cytotoxic TILs. Appropriate positive and negative controls were included with each staining run. Diaminobenzidine was used as the chromogen, and hematoxylin as the counterstain.

Selection of areas for evaluation

Immunostained slides were assessed in representative viable tumor areas. Regions showing extensive necrosis, hemorrhage, crush artifact, or non-representative tissue were excluded from scoring. Wherever feasible, the staining pattern was recorded in relation to intratumoral and perivascular areas, particularly for CD8-positive lymphocytes.

Interpretation and scoring of CD163

CD163 expression was evaluated in tumor-associated macrophages in representative high-power fields at 200× to 400× magnification. A case was considered positive when more than 5% of tumor-associated inflammatory cells showed membranous and/or cytoplasmic staining. In addition, CD163 expression was recorded using a semi-quantitative score from 0 to 3, where 0 = absent, 1 = weak, 2 = moderate, and 3 = strong expression. This scoring system allowed for both categorical and ordinal analysis of macrophage infiltration across tumor grades.

Interpretation and scoring of CD8-positive tumor-infiltrating lymphocytes

**Figure 1:**
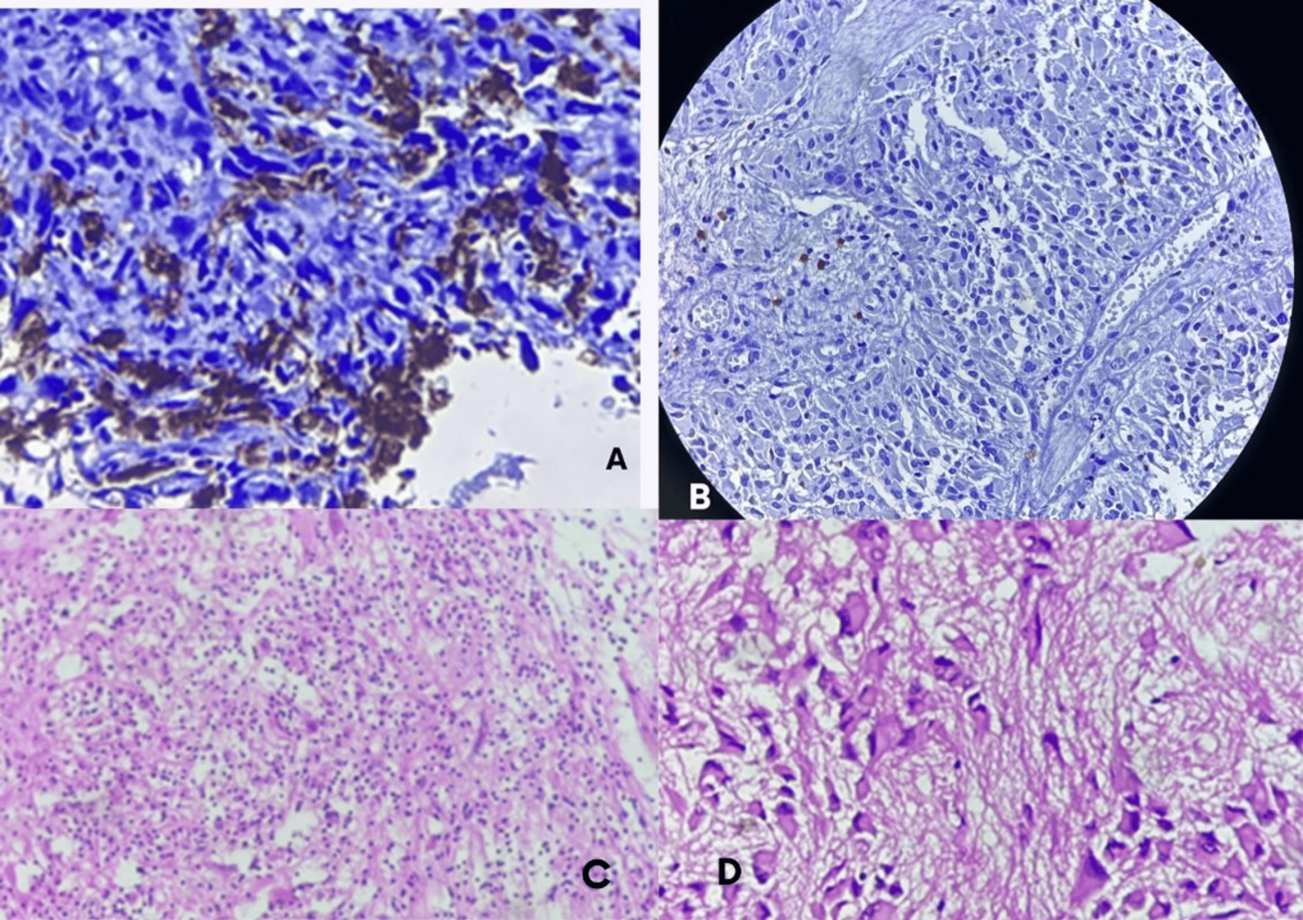
Immunohistochemical expression of CD163 and CD8 in low-grade and high-grade glial tumors Panel A: high-grade glioma showing dense CD163 expression. Panel B: CD8; high-grade ependymoma with sparse TILs (20x magnification). Panel C: low-grade glioma showing high TIL density (400x magnification). Panel D: high-grade glioma showing low TIL density (400x magnification) TIL: tumor-infiltrating lymphocyte

CD8-positive TILs were assessed across the entire section, and at least five high-power fields with the highest lymphocytic infiltrate were selected for evaluation. The proportion of CD8-positive lymphocytes was estimated relative to the tumor background, and the median value from these fields was taken as the case score. For analytical purposes, CD8 infiltration was categorized as low (10% or less) and high (more than 10%). The dominant distribution pattern was also recorded as intratumoral, perivascular, or both (Figure 1).

Study variables and outcome measures

The primary study variables were CD163 expression, CD8-positive TIL density, and WHO tumor grade. The primary outcome was the association of CD163 expression and CD8-positive lymphocytic infiltration with tumor grade. Secondary analyses included their relationship with histological markers of aggressiveness, such as necrosis and microvascular proliferation, as well as the reciprocal pattern between macrophage-rich and lymphocyte-poor tumor microenvironments.

Statistical analysis

Data were entered in Microsoft Excel and analyzed using appropriate statistical software. Descriptive statistics were used to summarize demographic and clinicopathological variables. Categorical variables were expressed as frequency and percentage. Associations between categorical variables were tested using the chi-square test or Fisher's exact test, as appropriate. Correlation of immune-marker expression with tumor grade was assessed using Spearman's rank correlation. Continuous variables, where applicable, were summarized using mean, standard deviation (SD), median, and range. A p-value less than 0.05 was considered statistically significant.

## Results

Clinicopathological profile

A total of 50 histopathologically confirmed glial tumors were analyzed. The study cohort showed a male predominance, and the overall mean age was approximately 42 years. Grade IV tumors formed the largest subgroup, and, when grouped broadly, high-grade gliomas outnumbered low-grade lesions, indicating that the cohort was weighted toward biologically aggressive tumors (Table 1).

**Table 1:** Clinicopathological profile of the study cohort (n = 50) SD: standard deviation

Variable	Value
Total cases, n	50
Mean age, years, mean ± SD	42.7 ± 16.1
Male sex, n (%)	29 (58.0%)
Female sex, n (%)	21 (42.0%)
WHO grade I, n (%)	4 (8.0%)
WHO grade II, n (%)	14 (28.0%)
WHO grade III, n (%)	12 (24.0%)
WHO grade IV, n (%)	20 (40.0%)
Low-grade tumors (I-II), n (%)	18 (36.0%)
High-grade tumors (III-IV), n (%)	32 (64.0%)

CD163 expression in relation to WHO tumor grade

CD163 expression was detected in the majority of tumors and showed a clear grade-dependent pattern. Higher-grade gliomas demonstrated not only greater overall positivity but also a distinct shift toward moderate and strong staining intensity. Statistical analysis confirmed a significant positive correlation between WHO grade and CD163 score, and CD163 positivity was significantly more frequent in high-grade compared with low-grade tumors. These results demonstrate progressive enrichment of CD163-positive tumor-associated macrophages with higher tumor grades (Table 2).

**Table 2:** CD163 expression by WHO tumor grade CD163 scoring: 0 = absent 1 = weak 2 = moderate 3 = strong. Statistical analysis: Spearman rank correlation showed a positive association between grade and CD163 score (rho = +0.59, p < 0.001). Low-grade versus high-grade comparison for CD163 positivity was performed using Pearson's chi-square test with continuity correction (χ² = 8.25, p = 0.004) WHO: World Health Organization

WHO grade	Absent	Weak	Moderate	Strong	CD163-positive (>5%), n (%)
I (n = 4)	2	1	1	0	2 (50.0%)
II (n = 14)	6	3	4	1	8 (57.1%)
III (n = 12)	1	2	5	4	11 (91.7%)
IV (n = 20)	1	1	6	12	19 (95.0%)
Total (n = 50)	10	7	16	17	40 (80.0%)

CD8-positive tumor-infiltrating lymphocytes in relation to WHO tumor grade

In contrast to CD163, CD8-positive TILs showed a declining pattern with increasing tumor grade. High CD8 infiltration was more often encountered in low-grade tumors, whereas most high-grade tumors exhibited low lymphocytic infiltration. This inverse relationship was statistically supported by Spearman's correlation analysis, and the difference between low-grade and high-grade gliomas also reached significance. Overall, these findings indicate progressive attenuation of cytotoxic lymphocytic infiltration as gliomas become histologically more aggressive (Table 3).

**Table 3:** CD8-positive tumor-infiltrating lymphocyte density by WHO tumor grade Statistical analysis: Spearman's rank correlation showed an inverse association between grade and CD8 category (rho = -0.33, p = 0.021). Low-grade versus high-grade comparison for CD8 infiltration was performed using Pearson's chi-square test with continuity correction (χ² = 3.97, p = 0.046) WHO: World Health Organization

WHO grade	High CD8 (>10%), n (%)	Low CD8 (≤10%), n (%)
I (n = 4)	2 (50.0%)	2 (50.0%)
II (n = 14)	7 (50.0%)	7 (50.0%)
III (n = 12)	3 (25.0%)	9 (75.0%)
IV (n = 20)	3 (15.0%)	17 (85.0%)
Total (n = 50)	15 (30.0%)	35 (70.0%)

Distribution pattern of CD8-positive lymphocytes

Evaluation of the localization pattern showed that CD8-positive lymphocytes were most commonly distributed in a perivascular pattern, whereas purely intratumoral infiltration was comparatively infrequent. This perivascular predominance became more evident in grade III and grade IV tumors, suggesting that in higher-grade gliomas, cytotoxic lymphocytes are more often spatially restricted rather than diffusely penetrating the tumor parenchyma (Table 4).

**Table 4:** Distribution pattern of CD8-positive lymphocytes by WHO tumor grade WHO: World Health Organization

Distribution pattern	Grade I (n = 4), n (%)	Grade II (n = 14), n (%)	Grade III (n = 12), n (%)	Grade IV (n = 20), n (%)	Total, n (%)
Intratumoral	1 (25.0%)	4 (28.6%)	2 (16.7%)	2 (10.0%)	9 (18.0%)
Perivascular	2 (50.0%)	7 (50.0%)	7 (58.3%)	13 (65.0%)	29 (58.0%)
Both	1 (25.0%)	3 (21.4%)	3 (25.0%)	5 (25.0%)	12 (24.0%)

Composite immune phenotype

When CD163 and CD8 were considered together, a composite immunosuppressed phenotype defined by strong CD163 expression with low CD8 infiltration was identified in approximately one-third of tumors. This phenotype was distinctly concentrated in higher-grade lesions and was uncommon in low-grade tumors. The association between this composite pattern and high-grade gliomas was statistically significant, supporting the concept that aggressive glial tumors are characterized by a macrophage-rich and lymphocyte-poor immune microenvironment (Table 5).

**Table 5:** Composite immunosuppressed phenotype by WHO tumor grade Statistical analysis: low-grade versus high-grade comparison was performed using Pearson's chi-square test with continuity correction (χ² = 8.26, p = 0.004) WHO: World Health Organization

WHO grade	Strong CD163 with low CD8, n (%)	Other immune pattern, n (%)
I (n = 4)	0 (0.0%)	4 (100.0%)
II (n = 14)	1 (7.1%)	13 (92.9%)
III (n = 12)	4 (33.3%)	8 (66.7%)
IV (n = 20)	12 (60.0%)	8 (40.0%)
Total (n = 50)	17 (34.0%)	33 (66.0%)

CD163 expression in relation to histologic markers of aggressiveness

Secondary analysis further showed that elevated CD163 expression clustered with morphologic hallmarks of aggressive glioma biology. Tumors exhibiting necrosis and microvascular proliferation were more likely to show moderate-to-strong CD163 staining, and both associations were statistically significant (Table 6).

**Table 6:** Association of CD163 expression with histologic markers of aggressiveness Statistical analysis: associations were tested using Pearson's chi-square test with continuity correction. For necrosis: χ² = 6.87, p = 0.009. For microvascular proliferation: χ² = 5.07, p = 0.024

Histologic variable	Cases with feature present, n (%)	Moderate/strong CD163 among present cases, n (%)	P-value
Necrosis	20 (40.0%)	18/20 (90.0%)	0.009
Microvascular proliferation	18 (36.0%)	16/18 (88.9%)	0.024

## Discussion

The present study findings are strongly aligned with the existing literature. Large transcriptomic and translational analyses have shown that CD163 expression increases with glioma grade and is particularly enriched in glioblastoma, IDH-wild type tumors, and mesenchymal phenotypes, where it correlates with stromal activation, angiogenesis, checkpoint signaling, and poor survival [3]. Tissue-based immune profiling by Martinez-Lage et al. [4] likewise demonstrated that CD163-positive macrophages are the most abundant immune population in glioblastoma and are especially enriched in mesenchymal tumors. The current findings therefore reinforce the view that CD163 is not merely a descriptive macrophage marker but also a histologically visible surrogate of an immunosuppressive tumor state. The association observed here between moderate-to-strong CD163 expression and necrosis or microvascular proliferation further strengthens this interpretation, because these features are canonical hallmarks of biologically advanced glioma and are mechanistically linked to hypoxia-driven myeloid recruitment, vascular remodeling, and wound-healing programs as described in prior studies [6,10].

The inverse pattern seen with CD8-positive lymphocytes is also broadly concordant with prior observations, though with important nuance. Han et al. [7] reported that CD8-positive T-cell density declines with increasing glioma grade, a finding closely mirrored in the present series. In glioblastoma, Orrego et al. [5] similarly described an immune milieu that is macrophage-rich but relatively poor in lymphocytic infiltrates. Mechanistic work helps explain why this occurs. Ravi et al. [6] showed that a CD163-positive, HMOX1-positive myeloid subset drives IL-10-centered exhaustion programs in CD8-positive T cells, particularly around mesenchymal-like tumor regions. Tu et al. [8] further emphasized that glioblastoma-associated macrophages suppress CD8 trafficking, metabolic fitness, and cytotoxic competence through cytokine signaling, checkpoint activation, and extracellular matrix remodeling. Within that framework, the present data are biologically persuasive: as tumor grade rises, the immune compartment appears to become not only more myeloid-dominant but also less permissive to meaningful cytotoxic lymphocyte penetration.

The distributional pattern of CD8-positive cells adds another layer of relevance. In this cohort, perivascular localization predominated, especially in grade III and grade IV tumors, whereas purely intratumoral infiltration was infrequent. This is important because the mere presence of CD8-positive cells does not guarantee antitumor activity. Spatially restricted lymphocytes may be trapped at vascular or stromal boundaries and prevented from entering the tumor parenchyma, where effector function would matter most. Emerging spatial studies support this interpretation. Najem et al. [11] showed that immune significance in CNS tumors depends not simply on abundance but on geospatial organization and interaction patterns. Sun et al. [12] likewise argued that the functional value of CD8-positive T cells is inseparable from their differentiation state and spatial position. Thus, the present finding of perivascular predominance in higher-grade tumors likely reflects ineffective immune access rather than robust immune surveillance.

At the same time, the current results should not be interpreted as implying that all CD8 infiltration is uniformly protective or that all macrophage infiltration is uniformly similar across tumors. This is where the discussion must remain intellectually honest. Some studies suggest that selected CD8 subsets, especially tissue-resident memory populations, may represent a more favorable immune contexture in glioblastoma, even when total CD8 counts are modest. Other work indicates that prognosis may depend less on absolute CD8 density and more on the balance between effector, regulatory, and myeloid compartments [7]. The present study is actually consistent with that more sophisticated view. The composite phenotype performed conceptually better than either marker alone because it captured immune imbalance more effectively. Aggressive tumors were not merely high in CD163 or low in CD8 independently; they were disproportionately characterized by the combination of both. That observation aligns with the broader immunobiologic principle that tumor progression is driven by networked immune dominance rather than single-cell abundance in isolation.

There are, however, areas where the present findings do not perfectly overlap with every prior report, and those discrepancies deserve explanation rather than dismissal. Martinez-Lage et al. [4] found that although macrophage infiltration varied markedly across glioblastoma molecular subtypes, CD8 differences were not statistically significant across subtypes. This appears less pronounced than the inverse grade-wise CD8 trend observed here. The discrepancy is plausible because subtype-based analysis within glioblastoma is not equivalent to grade-based analysis across the full spectrum of glial tumors. Molecular subtype comparisons ask whether different grade IV tumors vary in immune composition, whereas the present study asks whether low-grade and high-grade gliomas differ overall. These are biologically related but not identical questions. In addition, technical factors matter: tissue microarrays, multispectral quantification, whole-section assessment, different cutoffs, and different field-selection strategies can materially alter the measured lymphocyte density.

Another potential point of divergence comes from studies outside glioma, where high CD163 infiltration has not always signaled a poor state. In osteosarcoma, for example, Gomez Brouchet et al. [13] found that higher CD163 expression was associated with better survival. This apparent contradiction does not weaken the present findings because macrophage biology is context-dependent. CD163 identifies a phenotype that is often associated with alternative activation and immunoregulation, but its functional meaning is shaped by tissue lineage, cytokine environment, stromal architecture, and therapeutic exposure. Brain tumors are immunologically exceptional because of the blood-brain barrier, resident microglial populations, high steroid exposure, limited lymphocyte trafficking, and the distinctive metabolic conditions of the CNS microenvironment. Extrapolation from non-CNS malignancies should, therefore, be approached with caution.

The present study also diverges from an overly simplistic expectation that higher immune infiltration always means better prognosis. In many solid tumors, abundant CD8-positive TILs are indeed favorable, as shown in reviews by Contrera et al. [14], Rodrigo et al. [15], and Lin et al. [16]. Yet gliomas remain a special case because immune exclusion and dysfunction are common. Even when CD8-positive cells are present, they may be exhausted, spatially sequestered, steroid-suppressed, or counterbalanced by dominant myeloid suppression. This helps explain why the current findings place more emphasis on immune architecture and balance than on absolute lymphocyte count alone. It also explains why therapies that merely increase immune cell numbers may fail unless they also reverse suppressive macrophage programming.

From a clinical standpoint, the study has several implications. Firstly, CD163 appears to be a practical histopathological indicator of aggressive immune remodeling in glial tumors. Its rise across grades and association with necrosis and microvascular proliferation suggest that it may serve as an adjunct marker of hostile tumor biology, particularly in settings where molecular profiling is limited. Second, the combined CD163-high and CD8-low phenotype may have greater translational value than either marker in isolation because it more faithfully reflects an immunosuppressed ecosystem. Such a phenotype could help identify tumors that are less likely to respond to therapies that depend on intact cytotoxic immunity alone. Third, the predominance of perivascular rather than intratumoral CD8 localization in higher-grade tumors points toward immune exclusion as a structural problem, implying that therapeutic success may require not only checkpoint inhibition but also macrophage reprogramming, stromal remodeling, or strategies that improve lymphocyte penetration. Mechanistically and therapeutically, this is consistent with the growing rationale for targeting macrophage-associated pathways such as IL-10, CSF1-CSF1R, STAT3, and related myeloid checkpoints in combination with T-cell-directed interventions [6,8,17].

Limitations

The study also has some limitations. The sample size was modest and derived from a single center, which limits statistical power and generalizability. The cross-sectional design permits association but not causal inference, and survival analysis was not available; therefore, direct prognostic conclusions cannot be drawn from this cohort. Molecular stratification was not incorporated, particularly IDH status, O6-methylguanine-DNA methyltransferase (MGMT) promoter methylation, or mesenchymal subtype assignment, all of which are known to influence immune composition and would have strengthened the biological interpretation. CD8 assessment relied on density categories and distribution patterns but did not distinguish functional states such as progenitor exhausted, terminally exhausted, or tissue-resident memory subsets. Similarly, CD163 marks a major macrophage phenotype but cannot fully resolve the heterogeneity of glioma-associated myeloid populations. Finally, although whole-section evaluation is a strength in some respects, immunohistochemical scoring remains vulnerable to observer-dependent and sampling-related variation, especially in spatially heterogeneous tumors.

## Conclusions

The present study supports a biologically credible model in which glial tumor progression is accompanied by a gradual shift in the immune milieu, moving from relatively permissive low-grade states to macrophage-dominant, CD8-restricted, and spatially excluded high-grade microenvironments. The key finding is not simply that CD163 increases or CD8 decreases independently, but that their imbalance becomes more pronounced with increasing tumor grade, culminating in a combined immunosuppressed phenotype predominantly observed in high-grade gliomas. This places the study in strong alignment with current neuro-oncologic thinking that glioma aggressiveness is closely linked to its immune architecture. In practical terms, CD163 and CD8, particularly when evaluated together, provide a meaningful insight into glial tumor biology and may assist in guiding future risk stratification and immunotherapeutic approaches.
